# Intravital Imaging – Dynamic Insights into Natural Killer T Cell Biology

**DOI:** 10.3389/fimmu.2015.00240

**Published:** 2015-05-20

**Authors:** Pei Xiong Liew, Paul Kubes

**Affiliations:** ^1^Snyder Institute for Chronic Diseases, Cumming School of Medicine, University of Calgary, Calgary, AB, Canada

**Keywords:** natural killer T cells, intravital imaging, innate immunity, host–pathogen interactions, sterile inflammation

## Abstract

Natural killer T (NKT) cells were first recognized more than two decades ago as a separate and distinct lymphocyte lineage that modulates an expansive range of immune responses. As innate immune cells, NKT cells are activated early during inflammation and infection, and can subsequently stimulate or suppress the ensuing immune response. As a result, researchers hope to harness the immunomodulatory properties of NKT cells to treat a variety of diseases. However, many questions still remain unanswered regarding the biology of NKT cells, including how these cells traffic from the thymus to peripheral organs and how they play such contrasting roles in different immune responses and diseases. In this new era of intravital fluorescence microscopy, we are now able to employ this powerful tool to provide quantitative and dynamic insights into NKT cell biology including cellular dynamics, patrolling, and immunoregulatory functions with exquisite resolution. This review will highlight and discuss recent studies that use intravital imaging to understand the spectrum of NKT cell behavior in a variety of animal models.

## Introduction

Modern advances in technology have provided a plethora of *in vitro* and *ex vivo* methods to investigate the molecular systems and cellular functions of immune cells. These advances have resulted in significant insights into biological processes at the cellular level and deciphered multiple complex signaling pathways. Nevertheless, the most relevant experimental conditions in which to observe and document these biological processes remain the live animal. The use of intravital microscopy (IVM) provides such a view into the lives and dynamic interactions of diverse immune cell populations in various tissues and organs. Importantly, IVM is performed under experimental conditions which closely resemble the natural environment. As cellular functions and behaviors are influenced by several factors such as shear forces, anatomical location, and extracellular components, absence of these factors could result in tremendously different outcomes in *in vitro* versus *in vivo* settings.

Historically, IVM was first employed in the nineteenth century with brightfield microscopy to visualize leukocyte trafficking in translucent tissues (1). In the last two decades, brightfield-based IVM has brought about important discoveries especially in molecular and biophysical mechanisms of leukocyte adhesion to endothelial cells (2, 3). However, this basic technique applying visible light could only visualize uniformly colorless cells sufficiently slowed by adhesion, which allowed them to be distinguished from rapidly flowing cells (4). The advent of fluorescence-based intravital imaging with modern optical imaging agents and equipment now opens up exciting possibilities for biological observations. Many immune cells can now be tagged with fluorescent probes to visualize their behavior in real time in a live animal. Other important additions to fluorescence-based IVM are the different varieties of confocal microscopes, which provide deep tissue imaging and better subcellular resolution by excluding out-of-focus light via point illumination and pinhole apertures (5, 6). For example, spinning disk confocal intravital imaging systems provide rapid image acquisitions at the expense of deep tissue imaging, and are extremely competent for dynamic observations of immune behavior and cell–cell interactions particular within the vasculature (7–9). In contrast, multiphoton microscope systems, which employ a pulsed infrared laser excitation to generate fluorescence, have allowed deep tissue imaging of cell–cell interactions up to 500 μm depth (10, 11).

In recent years, fluorescence-based confocal IVM systems have been employed to visualize immune cells in almost all types of tissues to address a variety of immunological questions. Natural killer T (NKT) cells are credited with modulatory roles in a wide variety of diseases, and there is great interest in employing these cells for therapy in diseases or as biomarkers for prognostic purposes. In this review, we will focus on how IVM as a tool has revealed novel insights into NKT cell dynamics and biology.

## NKT Cells – A Quick Primer

The name “NKT cell” was first conceived about 25 years ago, and was used to broadly define a subset of murine T lymphocytes that shared functional and phenotypic characteristics with the natural killer cell, including the NK1.1 (NKR-P1 or CD161c) surface marker (12, 13). Although the term NKT cell is now accepted and applied to these cells in both mice and humans, this definition is inaccurate and possibly misleading as NKT cells in certain mouse strains do not express NK1.1 due to the allelic divergence of NK1.1 genes (14, 15). To further complicate this classification, some conventional T cells have been described to spontaneously express NK1.1 after activation (16).

Around the time when NKT cells were identified, a novel process of presenting lipid antigens was discovered (17, 18). This antigen presentation process occurred through the MHC class I-like molecule designated as CD1 (cluster of differentiation 1) that includes CD1a–CD1e (19, 20). All of these CD1 molecules present lipids instead of peptides as antigens. While humans express all five CD1 genes, mice express only CD1d. In mammals, CD1d is highly conserved (21). Further studies in mice subsequently demonstrated that CD1d molecules presented lipids to invariant T cell receptor (TCR)-bearing cells, which also expressed NK1.1 (22–24). This finding led to the realization that NKT cells were reactive to CD1d, and that the invariant TCR α-chain and CD1d were essential for the development of NKT cells. These unique phenotypic characteristics are now used to define NKT cells. An excellent review highlights the detailed timeline of discoveries that contributed to the identification of NKT cells (12).

**Figure 1:**
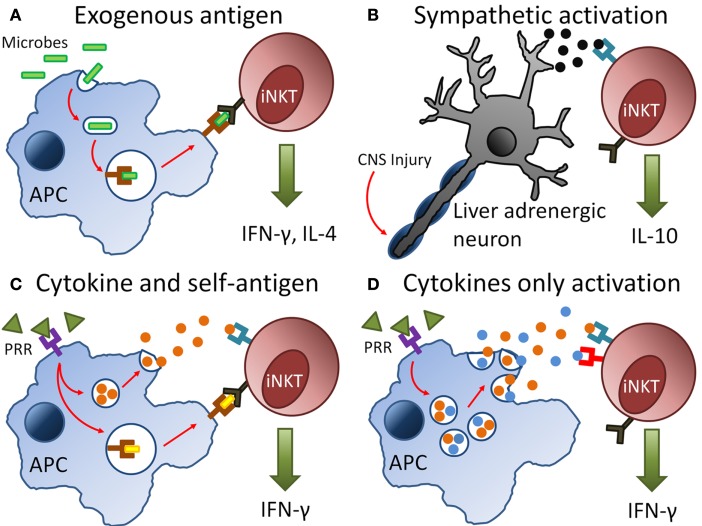
**Pathways of invariant natural killer T (iNKT) cell activation during infection and injury**. **(A)** Antigen-presenting cells (APCs) engulf invading microbes during infection and present exogenous antigens via CD1d molecules directly to the T cell receptor (TCR) on iNKT cells. **(B)** Injury to the central nervous system (CNS) results in signals transmitted via neurones and release of neurotransmitters such as noradrenaline. These neutrotransmitters bind to adrenergic receptors on iNKT cells resulting in their activation. **(C,D)** During infection and injury, pathogen-associated molecular patterns (PAMPs) or damage-associated molecular patterns (DAMPs) are released. These PAMPs and DAMPs bind to pattern-recognition receptors (PRRs) on APCs, which result in the production of inflammatory cytokines such as interleukin-12 and presentation of self-antigens on CD1d to the TCR of iNKT cells. The synergy of cytokine or self-antigen presentation contributing to iNKT cell activation depends on the type of injury or microbe involved during infection.

The discovery of the compound α-galactosylceramide (αGalCer) in 1997 contributed greatly to the understanding of NKT cells (25). This potent and specific lipid antigen, isolated from a marine sponge sample (likely from an infecting proteobacterium), was the first identified antigen for a specific population of NKT cells termed Type I NKT cells or invariant NKT (iNKT) cells. Through the use of CD1d tetramers loaded with αGalCer, iNKT cells in mice were discovered to express the invariant Vα14–Jα18 TCR α-chain paired with a β-chain biased toward Vβ2, Vβ3, and Vβ8 (26, 27). More than 80% of NKT cells were found to express these invariant chains. A similar TCR limited repertoire was found in human iNKT cells, which expressed Vα24–Jα18 paired with the Vβ11 chain (28). Due to large structural and functional similarities between the TCRs expressed by human and mice iNKT cells, αGalCer can bind to and activate iNKT cells from both species (29). In fact, this property has been taken advantage of by researchers to develop multimeric molecules with loaded synthetic αGalCer to identify iNKT cells *ex vivo* (30). These synthetic loaded tetramers are used in conjunction with anti-CD3 or anti-TCRβ antibodies to identify and enumerate iNKT cells in multi-parameter flow cytometry. In addition to αGalCer, a considerable number of exogenous ligands have been identified to activate iNKT cells (31). Further, self-derived endogenous lipids as well as the cytokines interleukin (IL)-12 and IL-18 have also been described to activate iNKT cells (32, 33). As iNKT cells can be activated by a range of exogenous and endogenous antigens and diverse inflammatory stimuli (Figure 1), they were found to be more important than initially realized in a variety of diseases (34, 35). Apart from Type I NKT cells, another subset of NKT cells has also been described (36–38). These Type II NKT cells recognize lipid antigens but express diverse TCR α- and β-chains (39, 40) and do not recognize αGalCer (41, 42). As this group of NKT cells cannot be identified through αGalCer-loaded CD1d tetramers, they are comparatively less characterized and understood (as compared to iNKT cells). This review shall focus mainly on findings discovered in mouse and human iNKT cell studies.

Although iNKT cells develop in the thymus, they are generally categorized as innate lymphocytes because iNKT cells exist in a poised effector state when they mature. Accordingly, mature iNKT cells are able to rapidly release large quantities of pro-inflammatory T helper type 1 (T_H_1) [(for example, interferon-γ (IFN-γ)] or T helper type 2 (T_H_2) (IL-4 and IL-10) cytokines within hours of activation (43, 44). In mice, resting iNKT cells contain preformed mRNA for both IFN-γ and IL-4 to allow swift cytokine production (45). These cytokines are able to transactivate other immune cells including neutrophils, NK cells, dendritic cells, and macrophages during an immune response (15, 46). Because iNKT cells are able to rapidly release substantial quantities of cytokines that can polarize the immune response, they are hypothesized to be important orchestrators of immunity. For example, activation of iNKT cells during infection results in the secretion of pro-inflammatory cytokines, which stimulates the developing immune response to fight off microbial invaders (47–49). A similar protective effect of iNKT cells is also observed during cancer (50, 51). On the other hand, iNKT cells can strengthen immuno-suppressive pathways during autoimmunity or ischemia–reperfusion injuries such as stroke (52, 53). In a mouse stroke model, there is increased sympathetic drive which induces iNKT cells to make more IL-10 and less IFN-γ (8). This leads to overall immuno-suppression but places individuals at a greater risk to infections. iNKT cells are therefore pivotal in shaping immune responses during diverse pathological states. An ongoing challenge is to unravel the factors that determine if iNKT cells facilitate or suppress an immune response.

Thus far, iNKT cells have been described to produce IL-2, IL-5, IL-6, IL-10, IL-13, IL-17, IL-21, tumor necrosis factor-α, transforming growth factor-β, and granulocyte monocyte-colony-stimulating factor (15, 54, 55). How does a single population of cells produce such a large variety of cytokines? The type and quantity of cytokine produced is influenced by several non-mutually exclusive factors. First, the quality of TCR signal (i.e., antigen signal strength and CD1d-binding kinetics) affects the cytokine profile. For example, use of different αGalCer analogs have been described to result in different ratios of IFN-γ/IL-4 produced (56–58). A similar phenomenon should occur with endogenous antigens compared to relevant foreign antigens. Second, targeting of antigen to different antigen-presenting cells will alter the pattern of cytokines made by iNKT cells (59, 60). Finally, functionally different subsets of iNKT cells have been described based on tissue localization and cell surface phenotype, which may promote different outcomes when iNKT cells are activated (15, 61).

## Imaging iNKT Cells

**Figure 2:**
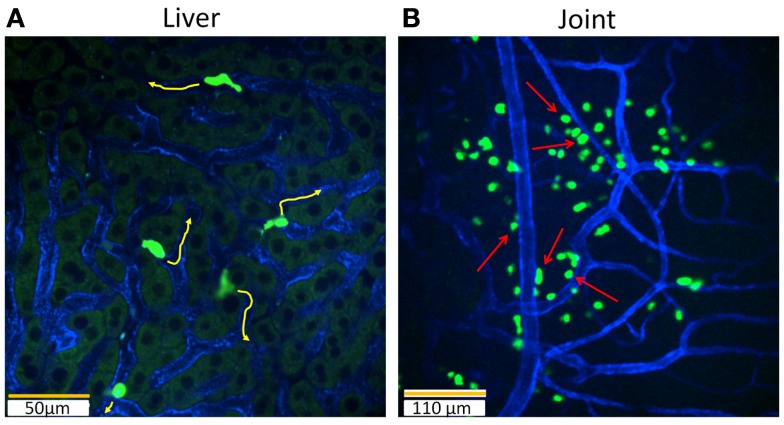
**Intravital imaging of iNKT cells in the liver and joints with *Cxcr6^Gfp/^*^+^ mice**. Still snapshots of videos recorded during imaging in a live animal are presented in: **(A)** iNKT cells (bright green) in the liver are intravascular and crawl on the luminal side of liver sinusoidal endothelial cells (blue) under basal conditions. When iNKT cells are activated, they no longer crawl and instead slow down their crawling phenotype or completely arrest. 20× objective, scale bar 50 μm. **(B)** In contrast, iNKT cells (bright green) in the joint are extravascular and line the capillaries (blue). These iNKT cells do not crawl and are stationary. During pathogen invasion in the joint, as in the case of *B. burgdorferi*, iNKT cells begin to crawl along vessel walls. Joint iNKT cells limit the dissemination of bacteria. Arrows: iNKT cells lining the vasculature. 10× objective, scale bar 110 μm.

There are a multitude of publications that describe the activation and cytokine production profiles of iNKT cells in mice and humans. However, their tissue distribution and dynamic behavior have only been brought to light recently. The capacity to visualize and observe iNKT cell behavior relies considerably on the labeling method. To date, no lineage-specific fluorescent antibody has been able to label iNKT cells. Isolating iNKT cells and staining them *ex vivo* with a fluorescent dye (for example, carboxyfluorescein diacetate succinimidyl ester) for adoptive transfer provides a manner to observe their behavior in an organ (62). However, this opens the possibility that cellular behavior may be altered by the potential artifact of cell isolation. So far, the best avenue is the use of genetically engineered knock-in mice where fluorescent proteins are inserted into a lineage-specific gene locus (63). Both mouse and human iNKT cells express high levels of the Cxcr6 chemokine receptor, which has been demonstrated to mediate the survival of iNKT cells in the liver (64–66). To image iNKT cells in a live animal, a mouse containing enhanced green fluorescent protein (GFP) inserted into the *Cxcr6* gene (*Cxcr6^Gfp/^*^+^) was generated (67). iNKT cells have been found to account for 75–80% of all GFP^bright^ cells in the liver. For the first time, the dynamic behavior of iNKT cells in different tissues and organs could be observed. Using IVM, hepatic iNKT cells were seen to crawl along the luminal side of liver sinusoidal endothelial cells without directional bias with an average speed of 10 μm/min (Figure 2A) (9, 67). This distinct behavior is unlike leukocyte behavior observed in post-capillary venules where leukocytes roll along continuous endothelium (3, 68). Detailed analysis of iNKT cell behavior in the liver demonstrated that iNKT cell crawling was random and independent of blood flow (67).

Resident iNKT cells are enriched in the liver, comprising up to 30% of all lymphocytes as compared to the thymus, lung, colon, bone marrow, spleen, lymph nodes, and blood (44). The cause for the higher frequency of resident iNKT cells in the liver is not completely clear; however, the adhesion molecule leukocyte function-associated antigen-1 (LFA-1, CD11a) has been suggested to be important in retaining iNKT cells in the liver (69). A tandem blockade of LFA-1 and its corresponding ligand, intercellular adhesion molecule 1 (ICAM-1), created a substantial rise in iNKT cells in blood and a reciprocal decrease in their number in the liver. Furthermore, LFA-1-deficient mice have notably reduced numbers of iNKT cells in the liver (70). Although we observed that the crawling phenotype of iNKT cells in liver sinusoids was not affected by LFA-1 and ICAM-1 antibodies, they did detach in collecting venules after treatment with blocking antibodies (71). Taken together, these data indicate that LFA-1 and ICAM-1 were perhaps necessary for interactions in larger vessels but not for crawling in sinusoids.

Previous studies have demonstrated that T cells arrest their movement when they encounter cognate antigen (72, 73). iNKT cells in the liver exhibit a similar behavior; when αGalCer was injected intravenously, crawling GFP^bright^ iNKT cells became stationary within an hour (67). Other studies have shown that hepatic iNKT cell arrest was correlated with iNKT cell activation (8, 9). Activation of iNKT cells via various mechanisms including CD1d, cytokines, or even neurotransmission all induce cell arrest within liver sinusoids. For example, synergistic effects between the inflammatory cytokines IL-12 and IL-18 resulted in the arrest of hepatic iNKT cells (74). In a mouse stroke model, norepinephrine release by the sympathetic nervous system during stroke caused rapid arrest of iNKT cells in the liver (8). Hepatic iNKT cells have been shown to express adrenergic receptors to receive neural signals (75). When iNKT cells are activated through cytokines or noradrenergic receptors, blockade of CD1d had no effect on the arrest of iNKT cells which suggests that classical antigen presentation through CD1d did not play a major role in arrest and activation in these situations.

**Figure 3:**
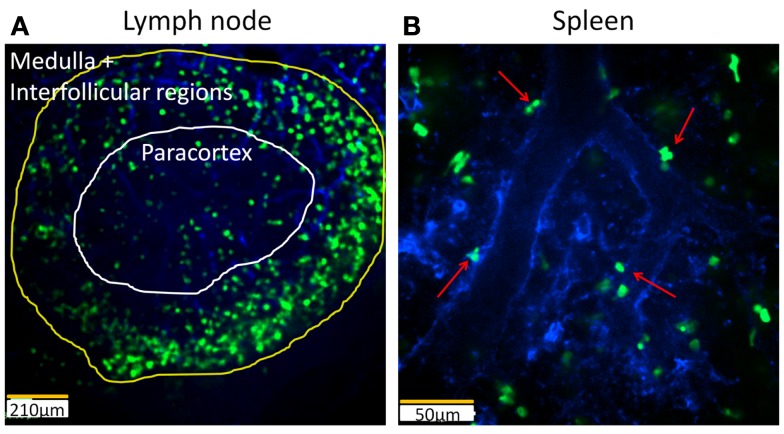
**Intravital imaging of iNKT cells in the lymph node and spleen of *Cxcr6^Gfp/^*^+^ mice**. **(A)** Still image demonstrating distribution of iNKT cells (bright green) in different regions of a lymph node. Vasculature of the lymph node is labelled with PECAM-1 (blue). The medullar and interfollicular regions are outlined in yellow while the paracortex is outlined in white. 10× objective, scale bar 210 μm. **(B)** Still snapshot of splenic iNKT cells (bright green) in red pulp of spleen reveals that these cells are located outside the vasculature (blue). Arrows: extravascular iNKT cells. 20× objective, scale bar 50 μm.

Despite the fact that the frequency of iNKT cells in other organs than the liver is low, various studies have highlighted the importance of iNKT cells in these organs in response to blood-borne pathogens (76–78). The *Cxcr6^Gfp/^*^+^ mouse has been employed to study the spatial organization, behavior, and functional roles of iNKT cells in several organs including the joints, lymph node, spleen, and lung. In distinct contrast to the liver, intravital imaging of iNKT cells in joints revealed dramatic localization of these cells around the joint blood vessels but not inside the vessels (Figure 2B) (78). Joint iNKT cells also exhibited different behavior under basal conditions as they were stationary and non-motile. In the lymph node, iNKT cells are located in the medulla and the interfollicular region but mainly absent in the deep paracortex where naïve T cells reside (Figure 3A) (79). These iNKT cells are highly motile in the lymph node and actively communicate with resident subcapsular sinus macrophages. During systemic infection, resident macrophages produce IL-18 and complementary cytokines, which elicit an innate IFN-γ response from lymph node iNKT cells. On the other hand, iNKT cells were found to be widely distributed throughout the spleen, including B and T cell follicles in the periarteriolar lymphoid sheath, the marginal zone (MZ), as well as the red pulp (69, 76, 80). Dissimilar to the liver, iNKT cells were observed to be crawling outside the vasculature in the spleen (Figure 3B) (71, 76). Interestingly, the localization of iNKT cells changes in the spleen during infection or in the presence of cognate lipid antigens. Under these conditions, iNKT cells slow down or arrest, and are confined to the MZ where antigen-rich MZ macrophages and dendritic cells reside (76, 80).

Although there is evidence demonstrating that iNKT cells are important in allergy and airway inflammation, information on the anatomical distribution of iNKT cells in lung, their mechanism of activation, and role in lung diseases remain scarce (77, 81). Recent studies have attempted to address these questions. Two-photon fluorescence microscopy was employed to examine the localization of iNKT cells in a harvested lobe of a murine lung (82). They revealed that pulmonary iNKT cells mainly resided in the lung microvasculature. Upon exposure to aerosolized lipid antigen, iNKT cells mobilized and extravasated into lung tissue. Thomas et al. (69) showed that there was an approximate 10- to 20-fold enrichment of iNKT cells in blood drawn from the right or left ventricles of the heart as compared to peripheral blood. Another study suggests that some pulmonary iNKT cells can be long-lived (83). Using parabiotic mice, Bendelac and colleagues demonstrated that pulmonary iNKT cells do not recirculate between parabiotic mice pairs even after 30 days (69).

## Interactions between the Host and Pathogens

Although αGalCer was extracted from a marine sponge sample, the presence of this iNKT cell ligand in marine sponges was not linked to any physiological relevant function. This highly reactive glycolipid likely originated from a bacterium present inside the sample rather than the sponge itself as marine sponges are commonly colonized by α-proteobacteria such as *Sphingomonas* spp. (84, 85). Indeed, the physiologically relevant αGalCer-related compounds, α-glycuronylceramide, and α-galacturonosylceramide which are found in the cell wall of *Sphingomonas*, are strong and potent activators of iNKT cells (48, 49, 86, 87). In addition to ceramide-based compounds from *Sphingomonas*, glycerol-based lipids have been described to potently activate iNKT cells. These include α-galactosyldiacylglycerol from *Borrelia burgdorferi* and α-glucosyldiacylglycerol from *Streptococcus pneumoniae* (88, 89).

*Borrelia burgdorferi* is a spirochete pathogen that continues to spread in North America (90). This pathogen induces Lyme-disease, and delayed or inadequate treatment typically leads to disabling symptoms as the bacteria invade the joints, heart, and central nervous system (91). The liver functions as an important organ that is positioned to intercept disseminating pathogens in the blood (92). This interception is mediated by liver-resident intravascular macrophages (Kupffer cells), which ensnare pathogens from the blood stream. Visualizing iNKT cell activity in the liver showed dramatically altered iNKT cell behavior after *B. burgdorferi* infection (9). Instead of crawling through the liver sinusoids, iNKT cells formed clusters and arrested next to Kupffer cells that had captured *B. burgdorferi*. This clustering occurred as early as 4 h after exposure and could be inhibited by blocking the Cxcr3 receptor. Anti-CD1d antibody blocked the firm adhesion of iNKT cells to Kupffer cells as well as the activation of iNKT cells, which suggests that Kupffer cells were responsible for presenting antigens to activate iNKT cells. Intravital imaging during this process revealed that Kupffer cells phagocytose *B. burgdorferi* from blood for antigen presentation to iNKT cells, which then produce IFN-γ and other inflammatory cytokines. This activated the local hepatic innate immunity system to prevent bacteria dissemination.

The absence of iNKT cells in mice caused a remarkable 25-fold increase in *B. burgdorferi* burden in joints, but other organs did not have a similar burden, which indicated that iNKT cells in the joint microenvironment had a unique feature. This unique *in vivo* observation led investigators to question the functional significance of joint iNKT cells. Intravital imaging of the joint during *B. burgdorferi* infection revealed that extravascular iNKT cells interact directly with the spirochetes at joint blood vessel walls (78). This joint iNKT cell behavior was in distinct contrast to iNKT cells in the liver, which were oblivious to the pathogen in the absence of Kupffer cells. During this interaction, joint iNKT cells were no longer stationary but actively crawled along vessel walls toward the pathogen, perhaps due to complement activation. *B. burgdorferi* that interacted with iNKT cells subsequently died, which suggests that joint iNKT cells limit the dissemination of this pathogen into the joint. Indeed, absence of iNKT cells led to a large number of motile spirochetes outside the vasculature in the joint cavity of mice. It is worth noting that human joints had far fewer iNKT cells, and perhaps this may lead to the susceptibility of humans, but not mice, to *B. burgdorferi*-induced Lyme arthritis.

*Streptococcus pneumoniae* infection is a leading cause of morbidity and mortality in adults and children (93, 94). This encapsulated bacteria typically resides on the mucosal surface of the upper respiratory tract or the nasopharynx of humans and appears to be asymptomatic (95). However, if *S. pneumoniae* gains access to the sterile lower respiratory tract, it causes a potent inflammatory response that result in severe disease. In this situation, pulmonary iNKT cells are important in the protection of the host against an infection by *S. pneumoniae* (96). If iNKT cells are absent (in Jα18^−/−^ mice) following infection by *S. pneumoniae*, lower cytokine levels, less neutrophils, and increased bacteria burden were found in the lung. The iNKT cell-deficient mice also had increased mortality following *S. pneumoniae* infection. A recent paper demonstrated that this protective effect was dependent on recognition of a *S. pneumoniae* glycoplipid (89). The behavior of iNKT cells in the lung under basal and *S. pneumoniae* infection has not been fully elucidated due to challenges of imaging the lung. Moreover, it is unclear which immune cells are presenting *S. pneumoniae* glycolipids to iNKT cells. Clearly, it would be of benefit to examine the dynamics of iNKT cells in the lung under these different conditions.

Other naturally occurring microbial antigens including cholesterol ester from *Helicobacter pylori* (97), lipopeptidophosphoglycans from *Leishmania donovani* (98), and *Entameba histolytica* (99) can activate iNKT cells, but the antigenicity of these lipids are not well characterized and direct evidence of significant contribution of these lipid antigens during infection and disease remain elusive. Further study is necessary to determine their contribution to the activation of iNKT cells during these specific infections.

## The Balance between Regulating Inflammation after Tissue Injury Versus Host Defense

The inflammatory response is critical for host defense against invading pathogens. Known as sterile inflammation, inflammation also occurs when self tissue is damaged in the absence of infection (100). Akin to inflammation induced by microbes, sterile inflammation also results in recruitment of neutrophils, monocytes, and macrophages, and the release of chemokines and pro-inflammatory cytokines such as IL-1 (101). Sterile inflammation has been identified to underlie many medical afflictions such as burn injuries or ischemia–reperfusion injury in the heart, liver, and brain (102). Following the initial trauma, the outcome of these afflictions are immunosuppression and susceptibility of the host to subsequent infection. Some medical examples of these complications include patients with acute myocardial infarction, stroke, or major burn injuries. Systemically inhibiting inflammation in these conditions can lead to adverse infectious complications. With the ability to react to self or invasive pathogens, iNKT cells are the linchpins which can determine a favorable or detrimental outcome during inflammation in these conditions.

Able to respond to “self” lipid antigens, iNKT cells are able to regulate inflammation during tissue injury (46, 103). Several endogenous lipids have been proposed to activate iNKT cells, although identification of the primary endogenous lipid antigen is a subject of intense research (104–106). Nevertheless, during tissue injury and cell death, endogenous antigens can serve as danger signals to activate iNKT cells in the absence of exogenous ligands. The functional role of iNKT cells have been investigated in mouse models of burn injury. In a cauterization-induced corneal inflammation model, iNKT cell-deficient mice had increased neutrophil accumulation and higher levels of pro-inflammatory cytokines in the cauterized eye (107). In addition, lack of iNKT cells led to greater corneal edema and opacity. In this model, iNKT cells played an important role in curbing inflammation and maintained corneal clarity. A similar immunoregulatory effect was observed in a dorsal burn injury model where iNKT cells were found to mediate T cell proliferation after injury by producing IL-4 (108). Production of IL-4 by iNKT cells suppressed antigen-specific T cell delayed-type hypersensitivity after dorsal burn injury.

During stroke, intravital imaging revealed that norepinephrine release rapidly arrested and activated iNKT cells in the liver (8). Interestingly, this increased sympathetic drive induced activated iNKT cells to produce increased levels of anti-inflammatory cytokines such as IL-10, which led to post-stroke immunosuppression. This effect likely protects the brain from inflammatory damage (109) but also leaves the patient open to infection, which is a major cause of post-stroke death (110). In contrast, activating iNKT cells with the potent agonist αGalCer reduced bacterial infection after stroke (8). Collectively, these findings suggest that iNKT cell activation was not the determining factor that mediated immunosuppression after stroke but rather the adrenergic activation and modulation of iNKT cells resulted in a shift from pro-inflammatory to anti-inflammatory cytokine production. This also raises the possibility of therapeutically targeting iNKT cells in the liver to quench detrimental neuro-immunosuppression as long as it does not enhance inflammation in the brain.

## Concluding Remarks

There is no doubt that iNKT cells have a pivotal function in directing innate and adaptive immunity during diseases where their diverse effector repertoire can lead to varied outcomes ranging from promoting inflammation to immunosuppression. Despite recent advances in unraveling mechanisms of iNKT cell activation and a greater understanding of iNKT cell biology, more research to elucidate the interactions between iNKT cells and other leukocytes is still needed. Traditionally, visualizing the spatial distribution of iNKT cells and understanding the role of iNKT cells in context of other immune cells were through static snapshots of tissue sections. However, technological advances in fluorescence microscopy and maturation of IVM technology have revolutionized the iNKT cell research field, allowing us to image the behavior of these cells in different organs under basal and inflammatory conditions at high resolution.

Although the ability to accurately visualize cells in a live animal at microscopic scale provides exciting opportunities for biological observation, caution is still needed at this stage. Fluorescence IVM is dependent on labeling cell types, and current technology is restricted by an inability to label all cell types, structural components, and chemical mediators at the same time. Therefore, only visible cell–cell interactions can be observed. In addition, the reporter mice that are presently available for lineage specific cell types including the *Cxcr6^Gfp/^*^+^ mice are not entirely specific and in some organs like the intestines, the percentage of GFP-positive cells that are iNKT cells is <50%, which makes it impossible to specifically track these cells. Further, current limitations of IVM technologies do not allow high-resolution imaging of all tissues and for those that can be visualized, it may not be possible to image deep into the tissue. Ongoing improvements to IVM technology such as the use of multiphoton microscopes, far-red probes, and longer wavelength lasers would address some of these issues (5, 10). Finally, current IVM techniques do not allow large areas of the tissue to be scanned at quick speeds; this limits imaging to relatively slower dynamic processes for observation if macroscopic levels are desired. Nevertheless, a thorough understanding of the spectrum of iNKT cell behavior and mechanisms of action will occur as IVM technology improves. Understanding iNKT cell biology will ultimately determine our ability to successfully target iNKT cells for clinical applications.

## Outstanding Questions

1. What factors determine the outcome, inflammation versus immunosuppression, of iNKT cell activation during diverse pathological states?
2. What are the roles of iNKT cells in the context of other immune cells under basal and inflammatory states?
3. How does the location and behavior of iNKT cells in the liver differ from other organs (such as the spleen, lung, and intestine)? Are there any similarities?
4. How can an enhanced understanding of the spectrum iNKT cell biological behaviors be utilized to manipulate their function for clinical settings?
5. Are iNKT cell counts and roles altered during pathological states? Are they reversible and does it affect the therapeutic ability of iNKT cells?

## Conflict of Interest Statement

The authors declare that the research was conducted in the absence of any commercial or financial relationships that could be construed as a potential conflict of interest.
